# Book Review

**Published:** 1927

**Authors:** 


### Reviews of Books.

**Handbook for Queen's Nurses.** Pp. 78. London: The Scientific Press Ltd. 1924. Price 1s. 6d.—This handbook, which comes out with a preface written by Miss Peterkin, the well-known General Superintendent of the Queen Victoria's Jubilee Institute for Nurses, needs no further recommendation. A successful Queen's Nurse ought to be well informed on a very wide range of subjects, and this little handbook provides information on useful points concerning public health in all its branches, as well as many useful hints and methods of nursing the sick poor in their own homes.

**Advice to the Expectant Mother on the Care of her Health.** By F. J. Browne, M.D., D.Sc., F.R.C.S. Pp. 40. Paper covers. Edinburgh: E. & S. Livingstone. 1926. Price 6d. —While Assistant Physician in charge of the Antenatal Department of the Edinburgh Royal Maternity and Simpson Memorial Hospital, where the late Dr. Ballantyne initiated his pioneer work for the welfare of the expectant mother, the author has had exceptional opportunities for obtaining a practical knowledge of the supervision of pregnant women. This useful little book is the outcome of his experience in dealing with this class of patient. It is concise, practical, and up to date, though some pediatricians may disagree with his method of giving alternate breasts in four-hourly feeds. Without being an alarmist he lays due stress on the grave dangers incurred by neglecting the early signs of disease in pregnancy and parturition, and the importance of routine examinations in every case. This book can, with advantage, be placed in the hands of all expectant mothers irrespective of their class.

**Employment for the Tuberculous, Notes, etc., on Finding.** By W. Bolton Tomson, M.D. Pp. 27. Paper covers. Three figures. London: Baillière, Tindall & Cox. 1926. Price 1s. —This unassuming but excellent little volume is issued at a most opportune time. The necessity of finding suitable employment for tuberculous patients is becoming increasingly obvious, and the success of the Papworth Colony in Cambridgeshire has shown what can be done, given enthusiasm, unlimited or almost unlimited capital, and quite exceptional business ability. The Ministry of Health has encouraged the formation of Tuberculosis Care Committees in all areas, whose main function is the provision of suitable employment for patients after satisfactory sanatorium treatment. In addition, the Joint Tuberculosis Council has recently issued its report of a survey of the numerous schemes at present in existence. Dr. Tomson properly lays considerable stress on several points which, although well known to all workers in tuberculosis, are frequently ignored by ignorant but well-meaning amateurs. The first and most important of these is that no scheme can ever be entirely self-supporting. Even the most successful must be subsidised. The second fact is that the tuberculous patient must work with others similarly affected, or he must be his own master. It is essential that he be at liberty to work when he can and rest when he ought, and any work that is found for him must conform to this condition. In the later chapters Dr. Tomson describes in detail what has been done in Hastings for a number of patients. He shows that it is impossible to deal with these patients in a mass, and that each case requires individual care and assistance. One particularly interesting chapter describes a successful attempt to find suitable employment for tuberculous women. This part of the book is of special value, as Dr. Tomson gives exact details of all that was done, including the cost—a most important point. Altogether, a most interesting and instructive little book, the careful study of which both by medical and lay members will add to the efficiency of any After-Care Committee.

**Functional Nervous Diseases.** By PAUL BOUSFIELD, M.R.C.S. Pp. 212. London: Wm. Heinemann Ltd. 1926. Price 6s.—This book presents within a small compass modern views on the nature and classification of functional nervous disorders. Part I. is devoted to a simple and useful exposition of dynamic psychology, avoiding the use of technical language, which frequently renders such books unreadable to the general reader. The concept of mental tension is well and simply propounded. Part II., which deals with the scope and methods of treatment, is good. It should be studied by those who vaguely imagine that the whole of modern psychotherapy is synonymous with psychoanalysis, for which they entertain a profound and prejudiced dislike. In Part III. of the book the author attempts to classify functional nervous disorders. The effect is not entirely successful. The distinction he draws between the psychoneuroses and the actual neuroses is by no means clear. His subdivision of types of hysteria is not very convincing, though pragmatically it may be useful. The author rightly emphasises the confusion that exists at the present time with regard to the term neurasthenia, pointing out that true neurasthenia is a rare condition. The name has become a dumping ground into which most of the little understood neuroses are piled. The book closes with a chapter on what he calls the organopsychic diseases, *e.g.* asthma and certain functional disorders of the intestines, which latter certainly need to be considered in their psychological aspects. The book is easy reading, and is certainly to be recommended.

**X-ray Diagnosis.** By J. MAGNUS REDDING, F.R.C.S. Pp. 227. 80 plates. London: Cassell & Co. Ltd. 1926. Price 21s.—This book is incorrectly described as "A Manual." It is really a Treatise, and a very good one, the type of detailed and careful work which we have long awaited from an English publisher. The author covers every branch of diagnostic radiography, closely correlating the clinical, anatomical, and skiagraphic findings, both normal and abnormal, but he wisely omits questions of technique except where necessary to the understanding of the methods employed. So good is the majority of the reading matter, that it throws into relief the poor quality of the illustrations, which in some cases, so far from being helpful, are a definite hindrance, and not worthy of the book at all. In spite of this, the volume is probably as valuable a general book of reference as is to be found in the English language at the present time. With really good illustrations, it would become a "standard work" on the subject.

**Chronic Rheumatic Diseases.** By F. G. THOMSON, M.D., M.R.C.P., and R. G. GORDON, M.D., M.R.C.P. Pp. 202. Oxford University Press. 1926. Price 8s. 6d.—This book epitomises very exhaustively our present-day knowledge of the so-called chronic rheumatic diseases, and demonstrates the fact that great progress has been made in their classification. Fibrositis has now become a definite clinical entity, but the condition called by the authors periarticular fibrositis will not, we think, be readily recognised by most medical men. The term rheumatoid arthritis is altogether abolished, to be replaced by *focal arthritis*, which includes also the gonococcal infection of joints. A sharp line of distinction is drawn between focal arthritis and osteo-arthritis, and included in the latter are *malum coxæ senilis* and the various forms of Charcot's joints.

Climacteric arthritis, which differs from many of the foregoing conditions, is described as a distinct disease. One of the most valuable parts of the book is that which deals with the common mistakes in diagnosis arising from such conditions as static deformities, new growths, diseases of the nervous system and the referred pains due to diseases of the viscera. Differential diagnosis of these conditions is entered into most clearly and fully ; some fifty pages are devoted to the principles of treatment, and it is noteworthy that drug treatment is dismissed in the short space of about two pages, the remainder being devoted to hydrology, physiotherapy, orthopædic and climatic treatment. The value of vaccines and of protein shock in the treatment of chronic rheumatic states is thoughtfully discussed, and the conclusion is arrived at that their value has been much exaggerated.

**Your Hair and Your Health.** By OSCAR L. LEVIN, M.D. Pp. ix., 163. London : Wm. Heinemann Ltd. 1926. Price 6s. net.—This is a practical book, in which Dr. Levin describes in the simplest and clearest manner the important part that the health of the body plays in the health of the hair. Apart from the constitutional diseases, the greatest enemies of hair growth are the local infections, so that a clean scalp is one of the best safeguards against loss of hair. The author quite rightly points out the absurdity of following barbers as the sole guides, when the only way to keep the hair is to keep the body healthy. He points out the advantages of bobbed hair, but at the same time warns its admirers that it is likely to be neglected, since its care is relatively easy. The book is full of interest, it is clearly printed and the few diagrams shown are good.

**Atlas of the History of Medicine.** Part I. : Anatomy. By Dr. J. G. DE LINT. London : H. K. Lewis & Co. 1926. Price 15s.—We have here 199 beautiful plates of ancient dissecting scenes, portraits of great anatomists and anatomical subjects, from Babylonian times down to the present day. With each plate is given a description of the scene or preparation or a short life of the anatomist shown in it, so that a complete survey of the growth of the science is placed before us in pictorial form. This collection of plates is quite unique. Nothing approaching it in richness exists elsewhere. The series gives not merely the story of how anatomy was gradually built up in the past, but also throws light on its new growth at the present time. As Dr. de Lint says, it is becoming the study of development, of the evolution, growth and relations of the various tissues and organs. Hence the portraits of the early microscopists, of Darwin, Huxley, Johannes Muller, and de Vries, emphasises this recent aspect of the science. The mediæval section is enriched with many of the recent discoveries of Sudhoff and Charles Singer. We could have wished for more of Leonardo da Vinci's drawings from the Windsor Castle collection. English anatomists indeed are well represented, considering that it is an international collection, and we are grateful for the large number of rare portraits of Dutch anatomists and the account of their work. The book is beautifully printed and issued in a convenient form.

**The Surgery of Gastro-Duodenal Ulceration.** By C. A. PANNETT, B.Sc., M.D., F.R.C.S. Pp. x., 154. Oxford Medical Publications. 1926. Price 10s. 6d. net.—This book of 152 pages gives a very good, modern, practical account of its subject, and can be cordially recommended. The references to the literature are frequent and discriminating, and the note of personal experience is very profitable. For the most part the conclusions drawn and the advice given are orthodox enough. The author gives point to his salutary warning that the surgeon ought not to intervene till the physician has had a chance, by relating cases in which operation was done too soon, the ulcer was too soft to be discovered, and another laparotomy was necessary later. He advocates brandy instead of tea and toast for the test meal. For gastric ulcer he advises sleeve resection in mid-gastric cases, and Billroth's (which he calls Péan's) operation for ulcers near the pylorus. For duodenal ulcers he performs a duodenectomy of the first part of the duodenum, and gives the technique. We do not think he has made out a conclusive case for this procedure. An interesting experience is related in which he operated within an hour or so for a perforated gastric ulcer still in shock, with a fatal result ; he believes that it would have been wiser to wait a little while for shock to moderate. He advises immediate operation for big hæmatemesis due to chronic ulcer, with a view to finding and ligating the bleeding point, using a blood-transfusion if necessary. Here, again, we are not convinced.

**The Inflammatory and Toxic Diseases of Bone.** By R. LAWFORD KNAGGS, F.R.C.S. Pp. xii., 416. 197 figures. Bristol: J. Wright & Sons, Ltd. Price 20s.—The author has spent his leisure time after retirement from active practice in the preparation and writing of this book. A large part of his work consisted in studying the specimens in museums and having microscopic preparations made of these. Therefore what ample time and painstaking care can do towards making a book good has been done. Included in the book is a description of different varieties of osteomyelitis, tuberculous and syphilitic disease, Charcot's joints, syringomyelia, yaws, osteo-arthritis, rickets, scurvy, osteitis fibrosa, osteomalacia, osteitis deformans, leontiasis ossea and osteogenesis imperfecta. The work is chiefly that of a morbid anatomist, the clinical aspect being much less completely dealt with, whilst treatment is referred to least of all. The subjects chosen for illustration and the character of the drawings are excellent, and the pictures alone make the book an invaluable work of reference for all those interested in the subject. Every section has a wealth of very good microphotographs, and these add greatly to the interest of the book. That the letterpress is not always as interesting as the pictures is perhaps the fault of the subject and not of the author. We cannot help regretting that a work of this kind could not have been written in conjunction with a clinical surgeon, who would have provided sections on X-ray diagnosis and on treatment, so as to present a more complete account of this difficult subject.

**Dental Materia Medica.** (Outlines of Dental Science, Vol. III.). By P. H. MARSDEN, M.Sc., Ph.C., F.C.S. Pp. 155. Edinburgh: E. and S. Livingstone. 1926. Price 7s. 6d.—This little volume is a very welcome addition to the various books now dealing with this subject. Without in any way attempting to bring forward a large text-book full of detail, the author has composed a book which should prove of considerable assistance to medical men, and perhaps more particularly to the dental specialist. The type matter is clearly written, and the chapters dealing with the different classes of drugs well arranged. With the minimum of difficulty one is enabled to find out the essentials of any drug mentioned, the book including as it does most of those in use. The author has skilfully included throughout the subject matter some helpful prescriptions. The dental student is somewhat apt to neglect the subject of drugs with their actions and uses. His training, tending to specialise on the mechanical side of his work, is inclined to make him think that materia medica is of very little concern to him. If he wishes to know much about the drugs he may be using he is forced to work through cumbersome text-books with much that is irrelevant to his special study of the oral cavity. This little book should prove of real assistance, the chapters devoted to prescription writing, mouth washes and tooth pastes being especially helpful.

**Handbook of Midwifery and Gynæcology.** By W. F. THEODORE HAULTAIN, O.B.E., M.C., F.R.C.S. Pp. viii., 316. 36 figures. London: Faber and Gwyer Ltd. 1926. Price 10s. 6d.—The author has set himself the task of compressing the whole of Midwifery and Gynæcology, including operative technique, into a small volume of some 300 pages. He has undoubtedly been highly successful. The whole subject-matter has been covered efficiently and is thoroughly up to date, and no important detail is missing. This success has only been obtained by the cutting out of every unnecessary word, so that the result is a book of undoubted value for an intensive revision course; but as the author remarks in his preface, it can in no way replace the fuller text books as the routine method of study in these subjects.

**Dental Anæsthesia.** (Outlines of Dental Science, Vol. I.) By G. F. RAWDON SMITH, M.D. Pp. 160. Illustrated. Edinburgh: E. and S. Livingstone. 1926. Price 7s. 6d.—The object of this small book is to furnish the dental student with a guide to the practice of anæsthesia as applied to his branch of the profession. It is founded on lectures delivered to dental students at the Liverpool University, and, in its 150 pages, it covers a considerably wider field than might have been considered necessary in a practical manual of this nature. For example, some of the theories as to the mode in which general anæsthetics act are included, and a short chapter is devoted to the chemical properties and method of administering chloroform. Either of these subjects seems to appear far too simple when dismissed so briefly, and, in the case of chloroform, one feels that a false sense of security may thus be imparted. The sections dealing with nitrous oxide, ethyl chloride, and ether are well written and contain those data which are most essential for successful administration. We consider that the chapter dealing with local and regional analgesia would be enhanced in value by the addition of more detail and some illustrations defining the sites of puncture. The book supplies an obvious want and should become very popular.

**Diseases of the Nose and Throat.** Third edition. By Sir ST. CLAIR THOMSON, F.R.C.P. Pp. xvi., 943. Illustrated. London: Cassell and Co. Ltd. 1926. Price £2 5s.—The scheme of this well-known text-book remains essentially the same as in the original edition. While a good deal has been omitted as being out of date, many new sections have been introduced and others extended to include recent additions to our knowledge. The section on local anæsthesia would have been valuable if the author had afforded the result of his long personal experience. The mention of borocaines with cocaine instead of among the substitutes is rather confusing. Sir St. Clair Thomson has been singularly successful in preserving balance and proportion throughout the volume, a task of very great difficulty when covering such an immense territory. As heretofore, his work will prove a reliable guide to diseases of the nose and throat for student and practitioner, while his colleagues in the speciality will appreciate this clear exposition by one who has attained world-wide eminence in this branch of medicine. We cannot close our notice without according praise to the splendid series of illustrations, to the excellent printing and production of the volume, and to the author's painstaking endeavour to render the work accurate and complete.

